# Trends in population-based incidence, diagnostics, and mortality of acute superior mesenteric artery occlusion

**DOI:** 10.3389/fsurg.2023.1334655

**Published:** 2024-01-03

**Authors:** Y. Soltanzadeh-Naderi, S. Acosta

**Affiliations:** ^1^ Department of Clinical Sciences, Lund University, Malmö, Sweden; ^2^ Vascular Centre, Department of Cardiothoracic and Vascular Surgery, Skåne University Hospital, Malmö, Sweden

**Keywords:** acute mesenteric ischemia, acute superior mesenteric artery occlusion, population-based study, trend, incidence, mortality

## Abstract

Acute occlusion of the superior mesenteric artery (SMA) results in lethal intestinal ischemia. Results from two previous population-based studies in Malmö, Sweden, suggest a decreasing incidence of acute SMA occlusion. This study aimed to evaluate trends in the epidemiology of acute SMA occlusion in Malmö. The report was a retrospective population-based study conducted from 2014 to 2019 on patients with acute SMA occlusion residing in Malmö municipality. Patient data were retrieved from Skåne University Hospital and postmortem examinations. Epidemiological data were compared to those of the two earlier studies, in particular to the one conducted from 2000 to 2006. Sixteen patients with acute SMA occlusion resided in Malmö municipality. The incidence of acute SMA occlusion significantly decreased from 5.4/100,000 person-years to 0.8/100,000 person-years. The ratio of acute SMA occlusion to non-occlusive mesenteric ischemia (NOMI) decreased from 12.5:1 to 0.9:1 (*p *<* *0.0001), the proportion of inhabitants aged 80 years or above in the population decreased from 6.0% to 4.3% (*p *<* *0.0001), and the autopsy rate decreased from 25% to 14% (*p *<* *0.0001). The in-hospital mortality rate decreased from 63% to 44% (*p *=* *0.14). The incidence of acute SMA occlusion seems to have decreased significantly in Malmö, probably due to high-resolution computed tomography angiographies being available around the clock to distinguish acute SMA occlusion from NOMI, a reduced proportion of elderly individuals, improved control of medical risk factors, and a decrease in autopsy rates.

## 1. Introduction

Acute mesenteric ischemia (AMI) is a condition in which the blood flow in the mesenteric vessels ceases, stopping the vascular supply to the intestines and causing intestinal ischemia. If not treated adequately, this may lead to intestinal necrosis and peritonitis (1). The most common cause of AMI is an acute thromboembolic occlusion of the superior mesenteric artery (SMA) (1–3). Other causes include non-occlusive mesenteric ischemia (NOMI) and superior mesenteric vein thrombosis (12). Diagnosing AMI has historically been challenging, and clear clinical and laboratory findings for diagnosing acute SMA occlusion are still lacking (4). Currently, the gold standard for diagnosis is computed tomography (CT) angiography: a modality that offers excellent sensitivity and specificity, with the advantage of being efficient and non-invasive (5–7). The most important treatment for survival in patients with acute SMA occlusion is intestinal revascularization, and in some cases, such as when transmural bowel necrosis is present, additional treatment with bowel resection is indicated (1).

The population of Malmö has previously been studied in relation to acute SMA occlusion in the years 1970–1982 and 2000–2006, with the incidence reported as 8.6 [95% confidence interval (CI) 7.6–9.7] per 100,000 person-years, decreasing to 5.4 (95% CI 4.3–6.4) per 100,000 person-years between the decades (89). Between 2000 and 2006, referred to as the 2000–2006 study throughout the rest of the paper, 49 out of 100 patients underwent active intervention: 20 underwent intestinal revascularization with or without bowel resection, and 29 patients underwent bowel resection only (9). The in-hospital mortality rate was 63%, with a cause-specific mortality rate of 3.0 per 1,000 deaths. The autopsy rate during the study period was 25%.

Recent studies have been carried out in other populations: a study from Helsinki, Finland, between 2006 and 2015 found 470 individuals diagnosed with acute SMA occlusion, with a total incidence rate of 3.1 per 100,000 person-years (10). The mean autopsy rate was 29%, lower in patients aged 70 years or above (18%). The study did not measure the in-hospital mortality rate but found that the 90-day mortality rate during this period was 83%. Another study from Estonia during 2016–2020 found 347 individuals with acute SMA occlusion, with an incidence rate of 5.2 per 100,000 person-years; 42% of patients underwent active intervention, and the in-hospital mortality rate was 69% (3). However, contemporaneous population-based reports on trends in incidence and mortality are lacking.

The aim of this study was to evaluate trends in population-based incidence, diagnostics, and mortality in patients with acute SMA occlusion.

## 2. Materials and methods

### 2.1. Reference and study population

Skåne University Hospital, with locations in both Malmö and Lund, is the sole hospital for acute and inpatient care in these cities. Vascular surgery services are, however, provided only in Malmö, which is the tertiary referral hospital for 1.8 million inhabitants in the southernmost part of Sweden. The reference population was derived by calculating the average number of inhabitants in Malmö for the years 2014–2019, resulting in 331,048 inhabitants per year, with 4.3% aged 80 years or above (Swedish Central Bureau of Statistics, SCB, www.statistikdatabasen.scb.se/goto/sv/ssd/BefolkningNy). During this period, the total number of deaths in Malmö was, on average, 2,651 per year (SCB, www.statistikdatabasen.scb.se/goto/sv/ssd/DodaManadReg). The total autopsy rate, including both clinical and forensic autopsies, for Malmö citizens was 13.6% (11).

Patients with acute SMA occlusion were identified by searching for patients admitted to Skåne University Hospital between January 1, 2014 and December 31, 2019 with the diagnostic codes K55.0 (acute vascular disorders of intestine) and I74.8 (embolism and thrombosis of other arteries) based on the International Classification of Disease, tenth revision (ICD-10). Patient records with these ICD-10 codes were studied through the medical journal system Melior. Patients diagnosed with intestinal ischemia at postmortem examination at the Department of Pathology in Malmö/Lund were acquired through the database Sympathy and a Laboratory Information Management System (LIMS) by conducting a search for ischemia using the topographic codes for the jejunum and ileum (T65) and the colon (T67). Individuals with intestinal ischemia diagnosed at the Department of Forensic Medicine in Lund were identified through a search by a statistician of the National Board of Forensic Medicine database using the diagnostic codes 444W (embolism and thrombosis of other specified artery) and 557A (mesenteric artery) based on the Swedish version of the ICD, ninth revision (ICD-9 RM). After removal of duplicates, a total of 236 patients were identified: 194 from the search at Skåne University Hospital, 34 from the search at the Department of Pathology, and 8 from the search at the Department of Forensic Medicine.

**Figure 1:**
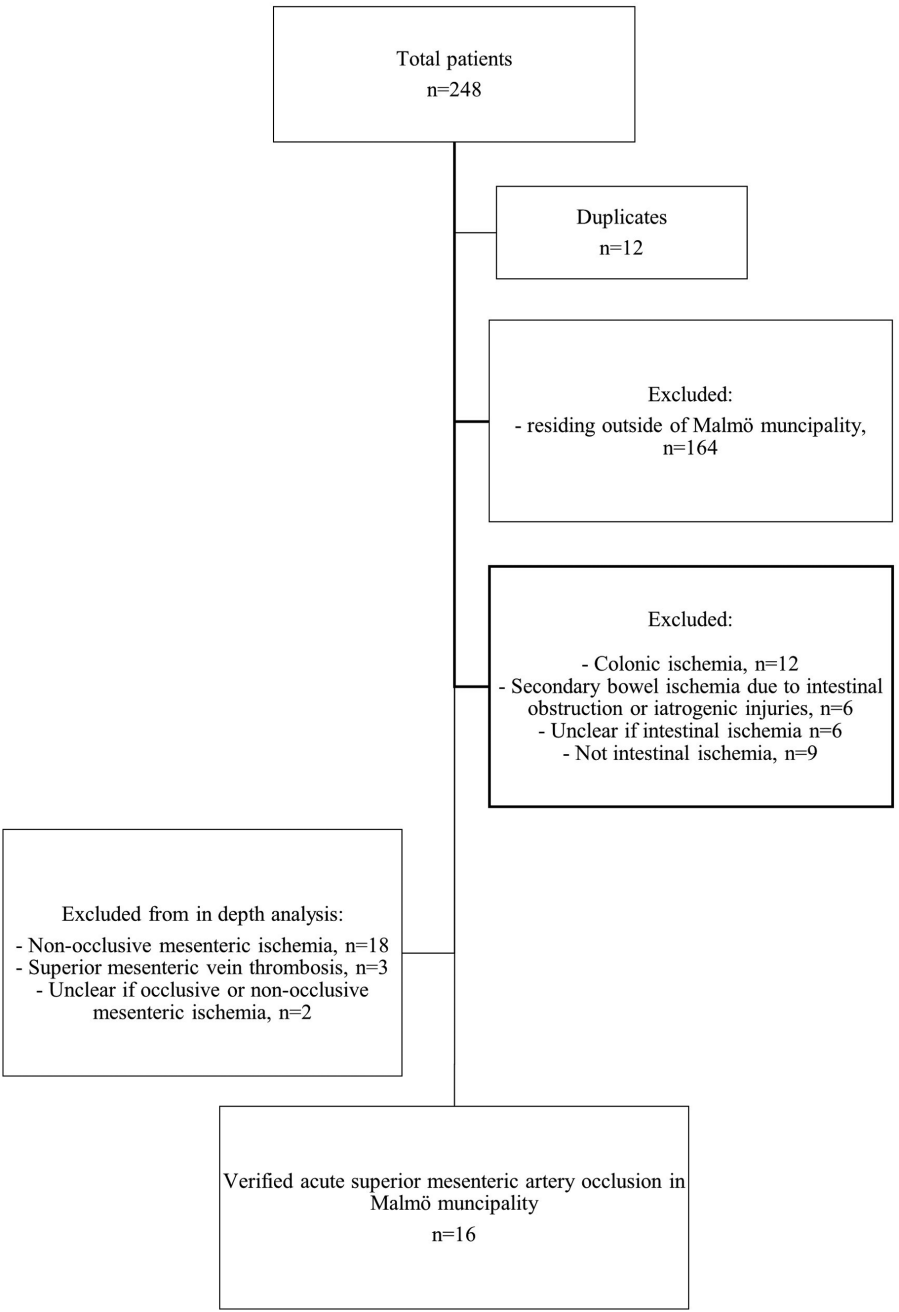
Flowchart representing the process of identification of the study population in Malmö municipality.

Patients who resided outside of Malmö municipality were excluded. Other exclusion criteria employed in conducting epidemiological calculations relating to acute SMA occlusion were NOMI, mesenteric venous thrombosis, uncertainty on occlusive or non-occlusive mesenteric ischemia, uncertainty on arterial or venous mesenteric ischemia, colonic ischemia, suspected AMI on clinical diagnosis, SMA dissection, secondary intestinal ischemia due to intestinal obstruction, iatrogenic injuries, uncertainty on intestinal ischemia, and absence of intestinal ischemia. After application of the exclusion criteria, the total number of patients with verified acute SMA occlusion in Malmö municipality was 16 (Figure 1).

### 2.2. Definitions

#### 2.2.1. The distinction between embolus and thrombus

The nature of native artery occlusion, embolic or thrombotic, was determined by the appearance of occlusion or high-grade stenosis (12) and the extent of atherosclerotic wall lesions in the SMA on CT angiography (13). Embolic occlusion often appears as an oval-shaped clot surrounded by contrast in a non-calcified arterial segment, whereas thrombotic occlusion usually appears as a clot superimposed on a heavily calcified occlusive lesion (13). The presence of synchronous embolism, atrial fibrillation, previous embolism, and no or inadequate anticoagulation therapy in patients with atrial fibrillation at the onset of AMI suggests embolism (13).

#### 2.2.2. Classification of etiologies of AMI

The classification of etiologies of AMI and the nature of the occlusion (thrombosis or embolism) was meticulously determined by a senior vascular surgeon (SA). All available CT images were carefully reviewed before determining the precise cause of AMI. The degree of SMA or celiac artery stenosis was measured by dividing the diameter of the narrowest part of the stenosis by the diameter of the normal artery distal to the stenosis and subtracting this value from 1; the value was expressed as a percentage. The degree of inferior mesenteric artery stenosis was dichotomized into occlusion/high-grade stenosis or not. On rare occasions, when CT had not been performed or was inconclusive, a combination of clinical findings, prior illnesses, surgical findings, and/or autopsy reports was used to assess the etiology. NOMI was defined as patent SMA on CT angiography or at autopsy with concomitant severe small bowel ischemia and/or severe colonic ischemia restricted to the right colon. The presence of low-to-medium-grade stenosis (12) of the SMA may coexist with NOMI. If there was no examination of the SMA but extensive gastrointestinal ischemia outside the supply of the SMA was present, with or without extraintestinal organ ischemia, the condition was labeled as NOMI (14). Acute-on-chronic mesenteric ischemia was defined as an emergency admission due to an acute ischemic event with intestinal infarction in a patient with a history of chronic mesenteric ischemia. Chronic mesenteric ischemia was defined as ischemic symptoms such as postprandial pain and weight loss considered to be caused by insufficient blood supply to the gastrointestinal tract with a duration of at least 3 months (1). Isolated left-sided colonic ischemia outside the primary supply of the SMA was classified as colonic ischemia (15) and not included in the definition of NOMI or AMI (1). Cases with no specification of localization of ischemia in the colon at autopsy were labeled as colonic ischemia. Patients who died from causes other than colon infarction but terminally developed moderate mucosal ischemia in the colon without small bowel ischemia found at autopsy were labeled as having colonic ischemia.

### 2.3. Statistics

A comparison of proportions of categorical variables between the 2000–2006 study (9) and the present study was conducted with a chi-squared test for *n* ≥5 and Fisher's exact test for *n* <5 using the GraphPad QuickCalcs website: www.graphpad.com/quickcalcs/contingency1/ (accessed December 2022). Incidence was expressed as individuals per 100,000 person-years. Cause-specific mortality was expressed as deaths from acute SMA occlusion per 1,000 deaths in total. Confidence intervals were calculated by assuming a Poisson distribution and using a normal distribution for *n* ≥15, and using the exact method for smaller numbers. A significant *p* value was defined as one below 0.05.

### 2.4. Ethics

The study was approved by the Swedish Ethical Review Authority (Dnr 2022-00812-01). All research was performed in accordance with relevant guidelines and regulations. Informed patient consent was not obtained since the study was retrospective, all the data had already been collected, and it is likely that most patients were deceased when the study was conducted. Moreover, all data are presented on a group level such that no individuals can be identified.

## 3. Results

### 3.1. Etiologies, treatment, and mortality rates of subtypes of AMI

**Figure 2:**
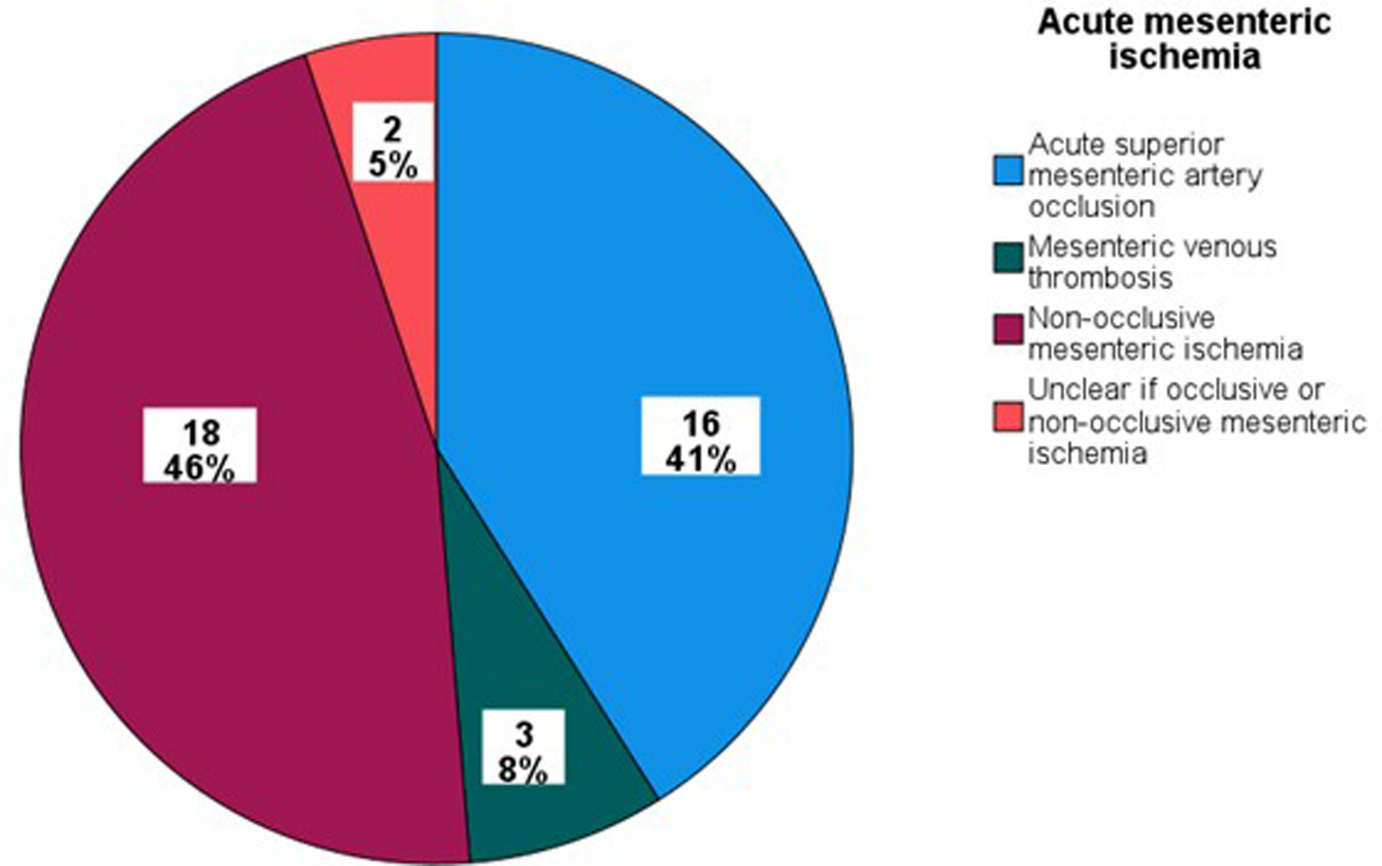
Etiologies of acute mesenteric ischemia in Malmö municipality.

Of the 236 individuals identified between 2014 and 2019, 39 suffered from AMI in Malmö municipality: 16 (41%) had verified acute SMA occlusion, 18 (46%) had NOMI, two (5%) suffered from either occlusive or non-occlusive mesenteric ischemia, and three (8%) suffered from mesenteric venous thrombosis (Figure 2). The overall incidence of AMI was 2.0 (95% CI 1.3–2.6) per 100,000 person-years. The incidence rates, treatment, and in-hospital mortality rates of subtypes of AMI are presented in Table 1. Among 18 patients with NOMI, 17 underwent CT angiography, showing no SMA stenosis in 16 and SMA stenosis <30% in one; and no celiac artery stenosis in 12, celiac artery stenosis ≥50% in three, and celiac artery stenosis <50% in two. The overall incidence of NOMI was 0.90 (95% CI 0.49–1.32) per 100,000 person-years [0.45 (95% CI 0.21–0.86) for men and 0.45 (95% CI 0.21–0.86) for women].

**Table 1:** Population-based incidence rate, active treatment, and in-hospital mortality rate of acute mesenteric ischemia.

	Acute embolic SMA occlusion (*n* = 7)	Acute thrombotic SMA occlusion (*n* = 9)	NOMI (*n* = 18)	Mesenteric venous thrombosis (*n* = 3)
Incidence^a^, *n* (95% CI)	0.35 (0.14–0.73)	0.45 (0.21–0.86)	0.90 (0.49–1.32)	0.15 (0.03–0.44)
Active treatment methods^b^	Endovascular aspiration (*n* = 3) Local intra-arterial thrombolysis (*n* = 5) Mechanical thromboembolectomy (Angiojet™; *n* = 3) Open SMA embolectomy (*n* = 1) Small bowel resection (*n* = 2) Large bowel resection (*n* = 1) Open abdomen (*n* = 1)	Stent graft/stent (*n* = 4) Local intra-arterial thrombolysis (*n* = 2) Mechanical thrombectomy (Angiojet™; *n* = 2) Small bowel resection (*n* = 1) Large bowel resection (*n* = 2) Open abdomen (*n* = 1) Cholecystostomy (*n* = 1) Full-dose anticoagulation (*n* = 1) due to two different cancers	Small bowel resection (*n* = 2) Large bowel resection (*n* = 5)	Full-dose anticoagulation (*n* = 3) Small bowel resection due to stricture (*n* = 1)
In-hospital mortality rate (%)	2 (29)	5 (56)	16 (89)	0 (0)

*n*, number; CI, confidence interval; SMA, superior mesenteric artery; NOMI, non-occlusive mesenteric ischemia.

^a^ Number of individuals per 100,000 person-years.

^b^ The sum of surgical procedures may exceed the total number of patients due to multiple procedures in some patients.

The ratio of acute SMA occlusion to NOMI was 0.9:1, compared to 12.5:1 in the 2000–2006 study period (*p* < 0.0001).

### 3.2. Incidence of acute occlusion of SMA

Sixteen patients with verified acute occlusion of the SMA lived in Malmö, resulting in an incidence of 0.8 (95% CI 0.4–1.2) per 100,000 person-years (Tables 2, 3). Seven patients were 80 years old or older. Seven patients had an embolic occlusion. Eight had a thrombotic clot superimposed on a calcified lesion in the SMA, and one had a calcified obstruction without a visible superimposed clot on CT angiography. Among the nine cases of acute SMA thrombotic occlusion, concomitant celiac artery occlusion occurred in one, celiac artery stenosis ≥50% in four, celiac artery stenosis <50% in four, and occlusion or high-grade stenosis of the inferior mesenteric artery in seven. Three patients had acute-on-chronic mesenteric ischemia. Incidence rates for women and men were 0.8 (95% CI 0.3–1.6) per 100,000 person-years and 0.8 (95% CI 0.4–1.6) per 100,000 person-years, respectively. Eleven patients underwent active intervention: seven underwent intestinal revascularization only, and four underwent both intestinal revascularization and bowel resection.

**Table 2:** Epidemiological characteristics of patients with acute superior mesenteric artery occlusion in the population of Malmö.

Study years	2000–2006 (9)	2014–2019	*p*
Demographics			
Population of Malmö municipality (inhabitants)	267,000	331,048	
Inhabitants ≥80 years (%)	6.0	4.3	<0.0001
Frequency of autopsy (%)	25	14	<0.0001
Overall incidence rate^a^, *n* (95% CI)	5.4 (4.3–6.4)	0.8 (0.4–1.2)	
Median age (years)	82	77	
Primary mode of diagnosis, *n* (%)			
All	100	16	
Autopsy only	18 (18)	0 (0)	
Surgery	42 (42)	1 (6)	0.0050
CT	27 (27)	15 (94)	<0.0001
Angiography	2 (2)	0 (0)	
Clinical diagnosis	11 (11)	-	
Intestinal revascularization rate, *n* (%)	20 (20)	11 (69)	<0.0001
Endovascular revascularization, *n* (%)	14 (14)	10 (63)	<0.0001
Overall cause-specific mortality^b^, *n* (95% CI)	3.0 (2.3–3.8)	0.4 (0.2–0.9)	
In-hospital mortality rate (%)	63	44	0.14

CI, Confidence interval; CT, Computed tomography.

^a^ Number of individuals with acute SMA occlusion per 100,000 person-years.

^b^ Number of deaths from acute SMA occlusion per 1,000 deaths per year.

**Table 3:** Population-based incidence of acute superior mesenteric artery occlusion, including data on autopsy rate.

First author	Publication year	Population	Incidence^a^, *n* (95% CI)	Median age (years)	Females (%)	Study period	Embolism: thrombosis ratio	In-hospital mortality rate (%)	Active intervention^b^ (%)	Autopsy rate (%)
Acosta et al. (8)	2004	Malmö, Sweden	8.6 (7.6–9.7)	81	66	1970–1982	1.4	93	16	87
Acosta and Björck (16)	2003	Blekinge County, Sweden	5.3 (3.2–7.5)	84	67	1999–2002	5.0	62	63	19
Acosta et al. (9)	2010	Malmö, Sweden	5.4 (4.3–6.4)	82	75	2000–2006	1.1	63	49	25
Lemma et al. (10)	2022	Helsinki, Finland	3.1 (2.8–3.3)	81	62	2006–2015	0.8	83^c^	38	29
Kase et al. (3)	2022	Estonia	5.2 (4.7–5.8)	80	62	2016–2020	0.2	64	42	18
Soltanzadeh-Naderi	2023	Malmö, Sweden	0.8 (0.4–1.2)	77	50	2014–2019	0.8	44	69	14

n, number; CI, confidence interval.

^a^ Number of individuals with acute superior mesenteric artery occlusion per 100,000 person-years.

^b^ Intestinal revascularization and/or bowel resection.

^c^ 90-day mortality.

Two patients were excluded due to uncertainty regarding the pathogenesis being of occlusive or non-occlusive nature. If it is assumed that these cases were caused by acute SMA occlusion, as a sensitivity analysis, the incidence would be 0.9 (95% CI 0.5–1.3) per 100,000 person-years.

The proportion of patients aged 80 years or above in the Malmö population decreased from 6.0% (2000–2006) to 4.3% (2014–2019) (*p* < 0.0001) (Table 2).

### 3.3. Trends in diagnostics and intestinal revascularization rate in acute SMA occlusion

The primary mode of diagnosis has shifted from surgery to CT angiography for acute SMA occlusion: 42% of cases (*n* = 42) were diagnosed with surgery and 27% (*n* = 27) with CT between 2000 and 2006, whereas explorative laparotomy was used for diagnosis of 6% of cases (*n* = 1, *p* = 0.0050) and CT for 94% (*n* = 15, *p* < 0.0001) of cases between 2014 and 2019 (Table 2). Of the 15 patients diagnosed with CT, 10 underwent CT angiographies, two underwent CT with intravenous contrast in the venous phase, and three underwent CT without intravenous contrast as the primary mode of diagnosis. Two of the latter patients then underwent CT angiography, and one underwent explorative laparotomy. Ten patients (63%) underwent angiography with subsequent endovascular revascularization in the later time period (2014–2019), but angiography was not the primary mode of diagnosis. Autopsy was the primary form of diagnosis in 18% and 0% of cases, respectively. Autopsy rates decreased from 25% to 14% (*p* < 0.0001), and the intestinal revascularization rate increased from 20% to 69% (*p* < 0.0001) between the earlier and later time periods in Malmö.

### 3.4. Trends in mortality of acute SMA occlusion

The in-hospital mortality rate was non-significantly reduced from 63% in 2000–2006 to 44% (*p* = 0.14) in 2014–2019 (Table 2). Overall, the cause-specific mortality rate decreased from 3.0 (95% CI 2.3–3.8) per 1,000 deaths to 0.4 (95% CI 0.2–0.9) per 1,000 deaths between the two time periods.

### 3.5. Reports on population-based incidence and mortality of acute SMA occlusion including autopsy rates

The present population-based study was compared to other population-based reports (3, 8–10, 16) (Table 2). The highest incidence rate and mortality rate reported stem from Malmö in a report covering a time period from 1970 to 1982, including an autopsy rate of 87% in the population.

## 4. Discussion

In the present study, the incidence of acute SMA occlusion in Malmö showed a significant decrease compared to the 2000–2006 study: from 5.4 (9) to 0.8 individuals per 100,000 person-years. Even in the sensitivity analysis, the decrease in incidence to 0.9 per 100,000 person-years was significant. Several methodological and epidemiological factors likely contributed to this finding. Acute SMA occlusion was identified with greater accuracy by modern high-resolution multidetector row CT angiography in the present study. For example, in the 2000–2006 study (9), multidetector row CT was only performed from 2004 onward. In contrast to the 2000–2006 study, clinical diagnoses of acute SMA occlusion were no longer accepted as a confirmed diagnosis in the present study. This difference in inclusion contributed to the lower incidence observed in the present study. The high precision in the classification of etiologies of AMI resulted in a ratio of 0.9:1 between acute SMA occlusion and NOMI. In comparison, the ratio was 12.5:1 in the 2000–2006 study in Malmö (9), >15:1 in Helsinki between 2006 and 2015 (10), and 8.3:1 in Estonia between 2016 and 2020 (3). The low incidence of NOMI in these populations may be attributed to inconclusive CT performed with suboptimal protocols, perhaps resulting in an overestimation of the number of patients with acute SMA occlusion.

Improved primary and secondary prevention, such as lower smoking rates and better medication for cardiovascular disease, are likely to have contributed to the decrease in the incidence of acute SMA occlusion (17). In Skåne County, where Malmö is located, the smoking trend has been one of significant decline, from 18% in 2004–2007 to 11% in 2015–2018 (18). A study conducted in Malmö between 2008 and 2011 analyzed peripheral arterial disease in relation to treatment of its risk factors, which are similar to other atherosclerotic diseases (19). The authors showed that the use of medications including acetylsalicylic acid and statins had increased compared to 2000–2003. Novel oral anticoagulants are now widely available and have similar efficacy in preventing arterial embolism to vitamin K antagonists in atrial fibrillation (20) while being much more user-friendly. Medication with novel oral anticoagulants has been shown to be associated with high compliance and persistence (21), and the use of oral anticoagulation treatment has increased for poststroke patients with atrial fibrillation since the introduction of novel oral anticoagulants (22). Furthermore, a declining trend in mortality due to cardiovascular events and stroke has been observed in the last few decades (23). It is reasonable to believe that increased activity in the prevention of atherosclerosis, including lower smoking rates, medical treatment, a healthy diet, and physical activity (24), combined with anticoagulation treatment of atrial fibrillation, reduces the incidence of acute SMA occlusion.

In addition, the decreasing share of inhabitants aged 80 years or older and the decreasing autopsy rate from 2000–2006 to 2014–2019 have likely decreased the population-based incidence rate of acute SMA occlusion.

The cause-specific mortality rate decreased for acute SMA occlusion in the population of Malmö between the two time periods (2000–2006 and 2014–2019), which mainly reflects the lower incidence of the disease in the present study. Even though diagnosis made by CT improved significantly from 27% to 94% and the intestinal revascularization rate significantly increased from 20% to 69%, it was not possible to show a significant decrease in the in-hospital mortality rate in Malmö, despite the apparent large reduction in the in-hospital mortality rate from 63% to 44%. After implementation of a pathway and care bundle for the management of acute SMA occlusion in Helsinki, it was possible to show a reduction in 30-day mortality in the postintervention group (2018–2020) compared to the preintervention group (2014–2017), even in a multivariable regression model among those undergoing surgery (25). Even if the data were selected and stemmed from operated patients only, the unique study design and results will hopefully encourage more healthcare centers to follow their example.

A limitation of this study was the retrospective method, which carries an inherent risk of information bias. The study sample was limited, increasing the risk of type II statistical errors. Furthermore, the declining autopsy rate between 2000–2006 and 2014–2019, especially among the elderly (26), suggests that the incidence and mortality rates in the present study are underestimated. However, it is likely that more patients in current times are diagnosed alive with either CT angiography or explorative laparotomy in-hospital than previously (9), suggesting that the role of autopsy might be of less importance. Indeed, all patients included in this study were diagnosed in hospital. Similarly, in another contemporaneous population-based study on acute lower limb ischemia in Malmö (11), all patients appeared to have reached the hospital in time for diagnosis and none were diagnosed at autopsy, further strengthening this view.

A strength of the study was the thorough methodological assessment of every patient whose data were retrieved from the hospital's patient records and portmortem examination records. The high rate of use of modern CT angiography resulted in reliable images for accurate diagnoses. In addition, the existence of two older population-based studies in the same demographic area made it possible to determine trends in incidence and mortality rates, which is unique.

There is an ongoing international multicenter study, AMESI, in which the incidence of different forms of AMI per hospital bed or admission, outcome, patient characteristics, and key factors in care delay will be studied (27). However, this study will not provide accurate epidemiological population-based incidence or mortality rates according to the prospective study protocol, particularly since it only includes patients admitted to different European hospitals and does not include ex-hospital deaths due to AMI.

Having an updated study on the disease epidemiology of acute SMA occlusion in Malmö provides a unique opportunity to evaluate trends in incidence and mortality, which allows the detection of possible changes of value to both patients and caregivers: for instance, this may bring about the means to evaluate the potential effect of preventive measures taken within the population. With the improved diagnostic accuracy in modern times, it is likely that the homogeneity of this study population is greater compared to those of previous studies, increasing the accuracy of future reports on prognostic factors, of benefit for both future patients and future research on the disease.

In conclusion, the incidence of acute SMA occlusion seems to have decreased drastically, probably due to more accurate and more widely available high-resolution around-the-clock CT angiography for distinguishing patients with acute SMA occlusion from those with NOMI, better control of medical risk factors, a decreased proportion of elderly people, and a reduced autopsy rate.

## Data Availability

The raw data supporting the conclusions of this article will be made available by the authors, without undue reservation.
